# The effects of a multi-ingredient cognitive formula on alertness, focus, motivation, calmness and psychomotor performance in comparison to caffeine and placebo

**DOI:** 10.1186/1550-2783-11-S1-P45

**Published:** 2014-12-01

**Authors:** Kevin A Shields, Jeremy E Silva, Jacob T Rauch, Ryan P Lowery, Jacob A Ormes, Matthew H Sharp, Sean A McCleary, John Georges, Jordan M Joy, Martin Purpura, Ralf Jäger, Jacob M Wilson

**Affiliations:** 1 The University of Tampa, Tampa, FL, USA; 2 Increnovo LLC, Milwaukee, Wisconsin, USA

## Background

Most pre-workout supplements are based on the stimulant caffeine, containing anywhere from 100-300 mg of caffeine in a serving. While research has confirmed increased mental focus and acuity from the use of caffeine, stimulant sensitive individuals should assess their tolerance before using pre-workout supplements containing caffeine. Caffeine can have dose-dependent unwanted effects contributing to a nervous or anxious feeling that can keep athletes from staying focused and even sleeping well. Ingredients to increase the synthesis and release of neurotransmitters (Tyrosine, acetyl-L-carnitine, alpha-GPC), and blood flow to the brain (Gingko Biloba), offer neuroprotection (blueberry extract), and improve mental regeneration and reduce mental stress (L-Theanine) might offer a stimulant-free alternative to improve pre-workout cognition. Therefore, the purpose of the current study was to investigate the effects of caffeine and a stimulant-free pre-workout formula on alertness (A), focus (F), calmness (CAL), motivation (MOT), cognition (COG), reaction (R), motor reaction time (MR), memory (MEM) and vertical jump power (VJP).

## Methods

Five college-aged males volunteered to participate in this study and were randomly assigned to consume MindSet (Haleo Inc., San Diego, CA), Caffeine, and a placebo (rice flour) in a double-blind, placebo-controlled, randomized, crossover design. After baseline testing, subjects consumed one of the assigned supplements 30 minutes prior to testing. Tests were separated by a 48 hour wash-out period. All subjects participated in a variety of mental aptitude tests, visual reaction tests, and power output measurements. Mental aptitude tests (A, F, CAL, MOT) were measured on an interval scale. COG was measured as serial subtraction test; accounting for improvement in scores from pre and post testing. RT and MRT were measured through the use of Dynavision, and VJP was measured through Vertical Jump Test via Tendo Unit. Consent to publish the results was obtained from all participants.

## Results

Caffeine increased alertness (+19%), focus (+35%), cognition (+26%), memory (+11%), motivation (+10%) and vertical jump power (+1%), however, decreased calmness by 18%. MindSet increased alertness (56%), focus (58%), motivation (43%), cognition (26%), memory (+15%), vertical jump power (3%), and calmness by 6%.

## Conclusion

A stimulant-free multi-ingredient pre-workout formula can be as effective as caffeine in increasing cognitive functioning without the unwanted side-effects. The results of this pilot study should be confirmed in a larger scale study.

